# Characterization and Interpretation of the Aluminum Zone Refining through Infrared Thermographic Analysis

**DOI:** 10.3390/ma11102039

**Published:** 2018-10-19

**Authors:** Xiaoxin Zhang, Semiramis Friedrich, Bernd Friedrich

**Affiliations:** IME Institute of Process Metallurgy and Metal Recycling, RWTH Aachen University, 52056 Aachen, Germany; xzhang@ime-aachen.de (X.Z.); bfriedrich@ime-aachen.de (B.F.)

**Keywords:** zone refining, infrared camera, process control, zone length, crystal growth rate, aluminum

## Abstract

High purity metals are nowadays increasingly in demand to serve in electronic, photovoltaic, and target materials industries. The zone refining process is the most common way to achieve high purity in the final step of metal purification. Zone length and crystal growth rate are the main parameters that control the zone refining process. To determine these values, information about temperature profiles in the molten zone is necessary due to its direct correlation with these values. As the determination of this profile is not practically achievable in the present, the novel approach of applying an infrared (IR) camera during the zone refining of 2N8 aluminum is the focus of the investigation in this work. The whole temperature profile of the region near the molten zone was recorded by IR camera during the entire running process. The zone length and the crystal growth rate at each thermographic image shooting moment were successfully extracted by thermographic analysis. Results showed that both factors varied significantly, which is in contrast to the assumption in literature about their stability while running under constant input power and heater movement velocity, though noticeable purification took place in all of these experiments. However, the impurity concentration during refinement fluctuated remarkably. This was well-demonstrated by the tendency of variation in crystal growth rate attained in this work. These results provide a better understanding of the mechanisms of zone refining with an inductive heater and contributes to the optimization of the process.

## 1. Introduction

Zone refining is the most common method used in the production of most high purity metals (purity greater than 5 N). Its mechanism is based on the difference in solubility of impurities in the solid and liquid state. The purification takes place by redistributing the impurities at the freezing interface when one or a series of molten zone(s) move through a solid bar. Many experimental and theoretical investigations have been conducted in recent decades with focus on improving refining and/or production efficiencies [1,2,3,4,5,6,7,8,9,10,11,12,13,14,15]. Two general categories, as characterized by different emphases, can be summarized from these investigations. The first is in realizing the target within the frame of the impurity distribution profile by optimizing experimental parameters such as zone length, zone movement velocity, number of passes, etc. [1,2,3,4,5,6,7,8,9,10]. The second is a focus on knowledge of the molten zone, such as zone shape, zone length variation, and the position of the molten zone, by taking heat transfer and heat balance into consideration [11,12,13,14,15]. The shape of the freezing interface affects the dislocation density and the impurity segregation [12]. The zone position and zone length variation are closely correlated with the freezing interface movement (crystal growth) and the dissolving capacity of impurities in the molten zone. It is well known that zone length affects not only the ultimate concentration distribution of impurities but also the rate of its approach [6]. Applying longer zone lengths in early passes followed by shorter lengths in later passes [6,7,8,9], or constantly adjusting the zone length in an optimized relation with the position of the freezing interface [10], will be helpful in improving refining efficiency. However, the research on zone length shows that this is simultaneously affected by many experimental conditions such as heating power, zone movement velocity, crucible size and material, charging material, and the method of cooling. All of these result in the challenge of regulating the zone length. In the case of having high thermal conductive materials for both charge and crucible, a stable zone length is difficult to maintain, as in, for example, the, refining of cadmium in graphite crucibles [16]. However, graphite crucibles are commonly used as a heat susceptor during the refining of magnetic-non-coupling materials (such as aluminum) with induction heating zone melting equipment. Therefore, the molten zone of aluminum under such operational conditions is supposed to be unstable as well. However, it has been shown in our preliminary work [17] that the application of a thermographic analysis system (e.g., an infrared camera) can deliver a variety of new information to ease the complexities in the measurement of zone length. 

Based on these facts, the objective of this work is, firstly, to illustrate the molten zone movement process in horizontal zone refining of aluminum through thermographic analysis, and then to investigate the zone length variation and crystal growth rate under different combinations of power and zone movement velocity. Additionally, the relationship of impurity concentration distribution after refinement (refining efficiency) with crystal growth rate and zone length will also be studied in focus.

## 2. Experimental Procedure

The trials were conducted in industrial scale horizontal zone refining equipment provided with a single inductive heater capable of generating up to 45 kW with a maximum frequency of 10 kHz, while applying a graphite crucible of 100 cm in length. An infrared (IR) camera (InfraTec GmbH, Dresden, Germany) was deployed to record the refining process in the form of thermographic illustrations.

### 2.1. Experimental Design

**Figure 1:**
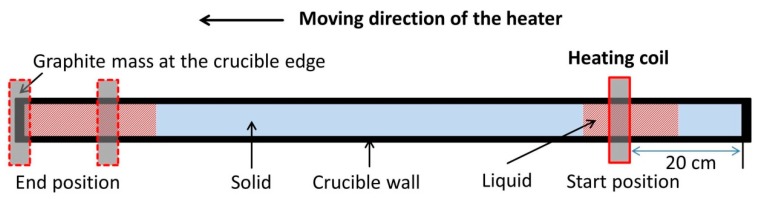
Sketch of the zone refining process.

Two different series of experiments, regarding the heating power and the heater movement velocity, were designed in this work, as shown in Table 1. As the zone refining process generally demands very low velocities in order to allow an effective segregation of impurities, the movement velocities of the heater were determined here as 1.2 mm/min as well as 0.8 mm/min (for the crucible used in this work, leading to periods of around 14 h/run and 21 h/run). Lower velocities were not examined due to the huge time consumption. A total of 9.8 kW of power is the minimum required to provide a molten zone along the bar, i.e., when the power is lower, the molten zone will disappear in the middle of the process or the metal cannot be melted. Due to an additional heating generated from the graphite (susceptor) mass at the end edges of the crucible while zone melting, the starting position of the heater was set at 20 cm after the edge. This assured a similar and stable heating effect along the investigated area. The experiments using a velocity of 0.8 mm/min ended at the moment when the melting interface of the molten zone arrived at the end of the crucible. In order to investigate the variation of zone length and crystal growth rate in the end section (unidirectional crystallization region), experiments using a velocity of 1.2 mm/min were run over that moment and finished until the heater was located at the end of the crucible. A sketch of the refining process is illustrated in Figure 1, where one pass was conducted for each experiment under 400 mbar Argon atmosphere. The initial material used was 2N8 aluminum initially doped with 0.1 wt % Fe as well as 0.1 wt % Si.

### 2.2. Data Analysis

#### 2.2.1. Thermographic Data Analysis

**Figure 2:**
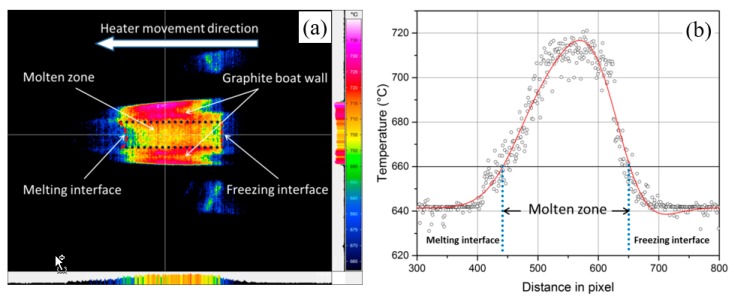
An exemplary thermographic image in zone refining of aluminum (**a**) and the corresponding temperature distribution profile in the molten zone along the bar (**b**).

A typical calibrated thermographic image at a certain time can be seen in Figure 2a. It clearly shows the temperature distribution along the graphite crucible and charge. The position of melting/freezing interfaces and the region of molten zone can also be distinguished. To calculate the exact interface positions, the temperature values along the molten zone were exported to form a plot of temperature against the distance in pixel as shown in Figure 2b. The region with a temperature higher than 660 °C can be defined as the molten zone when talking about pure aluminum. The length (in pixel) of this region and the positions (in pixel) of melting/freezing interfaces in relation to the beginning of the bar have been converted to length and position in cm using MATLAB (R2015a, MathWorks, Natick, MA, USA).

#### 2.2.2. Impurity Concentration Analysis

**Figure 3:**
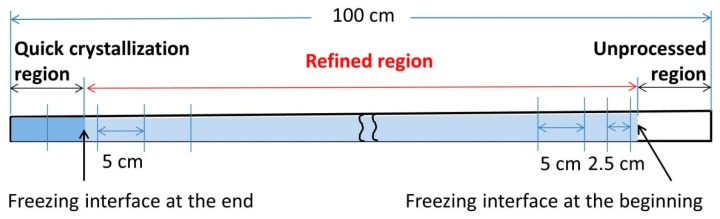
The form of the sample after refinement and the positions used for impurity concentration analysis.

A sample can be divided into three regions after refinement as shown in Figure 3. They are the unprocessed region, the refined region, and the quick crystallization region (while power is turned off). Numerous samples along the refined region as well as some along the area of the quick crystallization were taken and analyzed on the cross-section via the spark spectrometry method. The concentration distribution of Fe and Si in the refined region was assessed to reveal the refining efficiency. The impurity concentration in the quick crystallization region indicates the intensity of the accumulation of impurities. The attained concentration value at every position is defined as C_S_, and the ratio C_S_/C_0_ (where C_0_ is the initial concentration) represents the refining efficiency. The lower the value of C_S_/C_0_, the higher the refining efficiency.

## 3. Results and Discussion

### 3.1. Characterization of the Zone Refining Process

**Figure 4:**
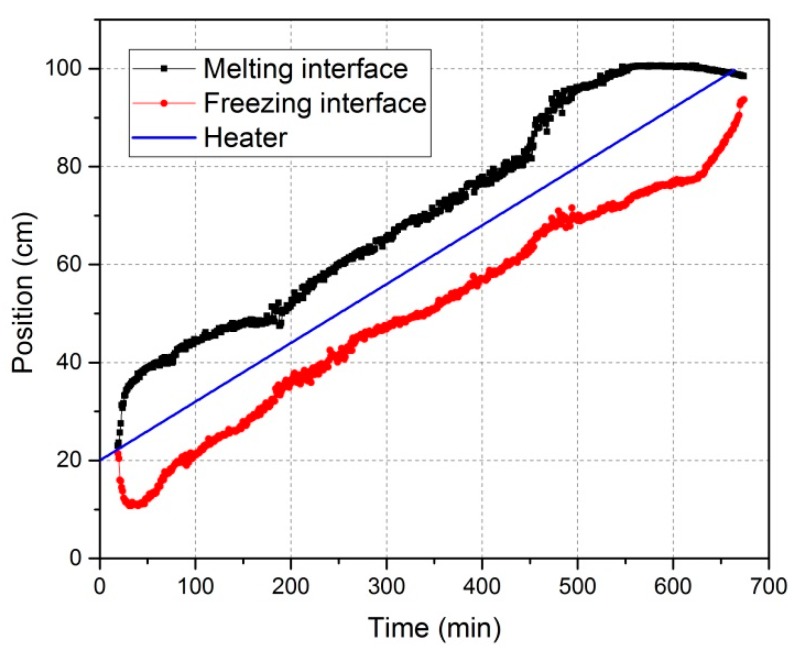
Positions of the melting interface, freezing interface, and heater against time for the experiment with 11 kW-1.2 mm/min, showing that the freezing- and melting interfaces moved nonlinearly.

**Figure 5:**
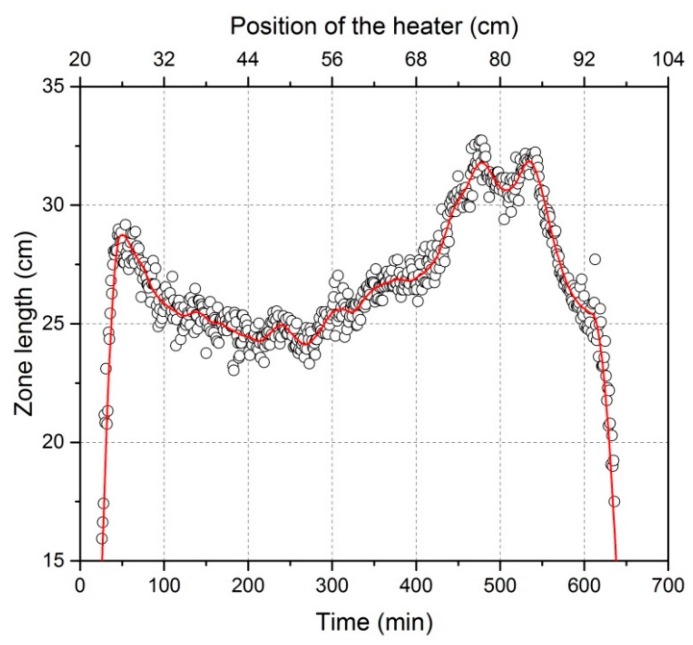
Zone length variation during the refining process derived from thermographic imaging for an exemplary experiment with 11 kW-1.2 mm/min.

Figure 4 illustrates the positions of melting and freezing interfaces during the continuous movement of the heater along the aluminum bar. The exact values of these positions have been attained from thermographic analyses. As a general rule, after the heater is switched on, a specific length of molten phase is formed around the heater. The two portions of this length, on the left or right side of the heater, were changing continuously while the heater moved along the bar. Consequently, the zone length, i.e., the difference between melting and freezing interfaces, was changing in the meantime (even in the case of consistent power and velocity), as shown in Figure 5. The zone length to be considered here corresponds to the time period of 40–550 min. This is because, in the first 40 min, the molten zone started forming and expanded along two opposite directions (see Figure 4) without crystallization, and the molten zone in the last 100 min was in the unidirectional crystallization region. Without changing the heating power, the zone length could be seen as quite large at both ends of the bar and relatively stable with smaller values in the middle of the bar. This is most probably due to the lower thermal dissipation through solid aluminum and inside the crucible when the molten zone is located close to one of both ends—with one available dissipation direction—in comparison to the locations in the middle of the bar with two available dissipation directions. After starting at the 20 cm position (see Section 2.1), the charge has a much shorter heat dispersion to one side—because the portion of the bar is much shorter—and a much longer one on the other side. However, these portions change when the heater moves to another end of the charge. Furthermore, it can be seen from Figure 4 that in the case of zone refining of aluminum, the heater was always located in the middle of the molten zone. This is attributed to the low Prandtl number of aluminum (low ratio of viscosity to thermal conductivity), preferentially resulting in symmetrical molten zones [11].

**Figure 6:**
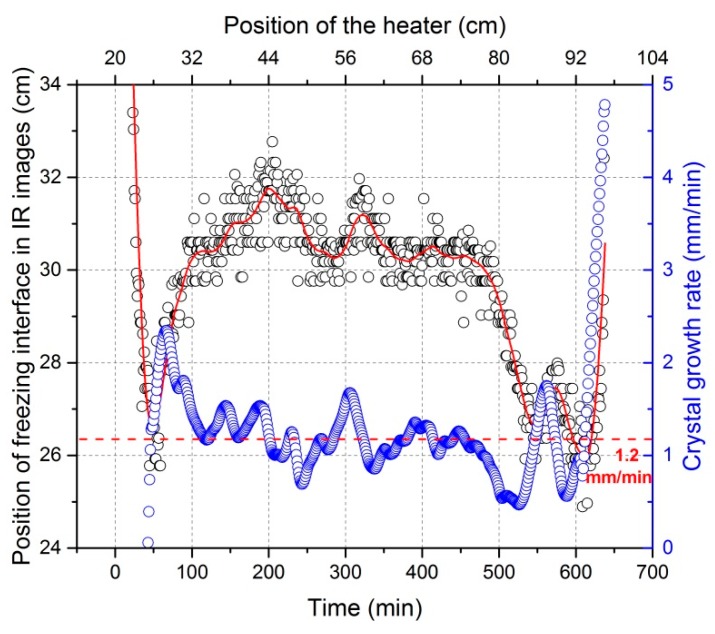
Variation in position of the freezing interface in thermographic images and the corresponding crystal growth rate against time in the case of moving the heating coil to the end of graphite crucible (the red dotted line represents the movement velocity of the heater).

Another measurement—attained indirectly via thermographic images at every image shooting moment—is the crystal growth rate. It is the summation of heater movement velocity and the freezing interface movement velocity in the thermographic image as the IR camera moves simultaneously with the heater. The position of the freezing interface in the thermographic image is actually data attained directly from an IR image, and its differential coefficient against time is its movement velocity in the image. It can be seen in Figure 6 that a significant variation of crystal growth rate takes place during the process. It should be pointed out that the tremendous growth rate at the end (after around 600 min in this experiment) was due to the significant reduction of induction heating. At this time, the molten zone had already arrived at the end of the crucible, i.e., the unidirectional crystallization appeared, and the heater was close to the end of crucible. Therefore, the graphite mass functioned as heating resources decreased as the heater moved forward. In addition, the mass of material was significantly reduced (a typical zone melting phenomena), leading to an accelerated solidification in this area. 

**Figure 7:**
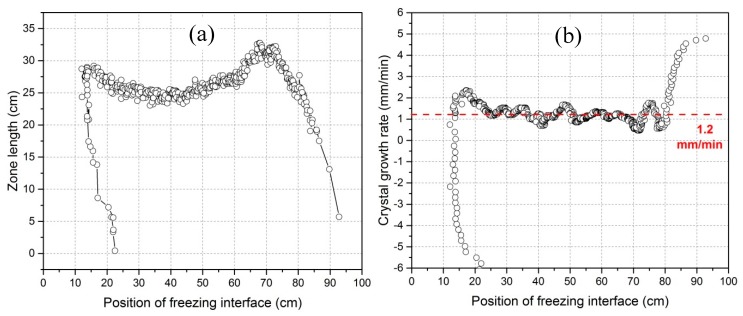
Zone length (**a**) and crystal growth rate (**b**) as a function of the position of the freezing interface.

The freezing interface is the place where the targeted purification occurs. In order to interpret impurity concentration profiles after refinement so as to improve the refining efficiency, a mathematical correlation of zone length and crystal growth rate with the freezing interface position is very desirable. As seen in Figure 4, the distance of the freezing interface from the beginning of the bar is not linearly proportional to time. Therefore, the relationship of zone length and crystal growth rate with freezing interface position is different from their relationship (see Figure 5 and Figure 6) with time. This result is shown in Figure 7.

### 3.2. Dependency of the Zone Length on Power and Heater Movement Velocity

**Figure 8:**
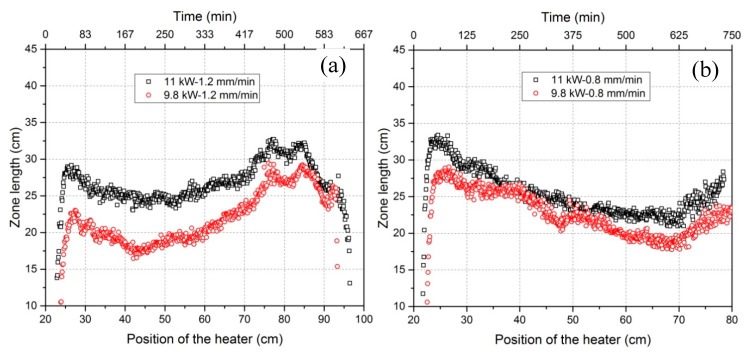
Zone length variation versus the position of the heater with a heater movement velocity of 1.2 mm/min (**a**) and a heater movement velocity of 0.8 mm/min (**b**).

**Figure 9:**
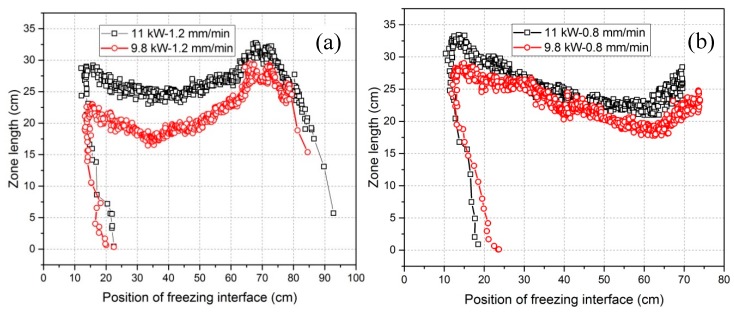
Zone length variation versus the position of the freezing interface for a heater movement velocity of 1.2 mm/min (**a**) and a heater movement velocity of 0.8 mm/min (**b**).

The relationships of the zone length with the position of the heater and the position of freezing interface are represented in Figure 8 and Figure 9. Both figures show similar zone length variation tendencies. The diagram of the zone length versus the position of the heater (or versus time) has so far been the only way to show the zone length variations when no IR camera is available. However, zone length versus the position of freezing interface—only possible when using an online IR camera—is more meaningful because it provides a novel chance to enable investigation of the effect of zone length on refining efficiency. In general, the zone length in all experiments showed a decreasing and then an increasing tendency. The selected characteristic values (listed in Table 2), however, were different. As a short, intermediate conclusion, higher power induces a greater zone length, as normally expected during zone melting process. In addition, lower heater movement velocities result in further delays in the position of the freezing interface while the zone length reaches the minimum level. In the case of a movement velocity of 1.2 mm/min, the zone length at the end was greater than that at the beginning, while the opposite was true for 0.8 mm/min. The percentage difference between maximum and minimum zone lengths in this work was quite large, ranging from 25% to around 36%. However, higher power seems to be beneficial in reducing this difference.

### 3.3. Crystal Growth Rate Variation

**Figure 10:**
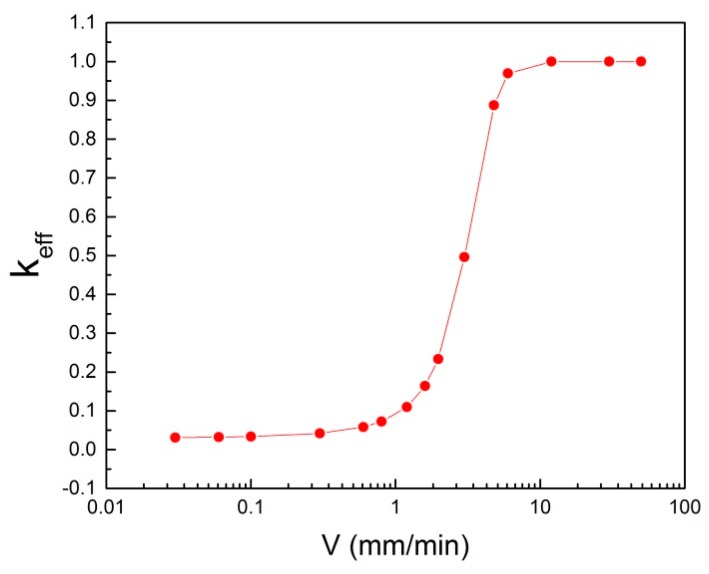
The relationship of k_eff_ with crystal growth rate for the impurity, Fe, in aluminum [18].

Zone length variation is the result of process window changes. The resulting changes of the freezing interface leads directly to a variation of the crystal growth rate, which strongly affects the effective distribution coefficient (k_eff_). This can be seen in Figure 10 as the dependency of k_eff_ of the impurity, Fe, in aluminum on crystal growth rate [18]. This correlation is based on the Burton-Prim-Slichter (BPS) model and shows a dramatic rise of k_eff_ while achieving the crystal growth rate of 1 mm/min. The following equation represents the effective distribution coefficient as well as the BPS model:

(1) keff=kk+(1−k)exp(−VδD)

where k is the equilibrium distribution coefficient, D is the impurity diffusion coefficient in the melt, δ the thickness of the diffusion boundary layer at the solid/liquid interface, and V is the molten zone movement velocity.

**Figure 11:**
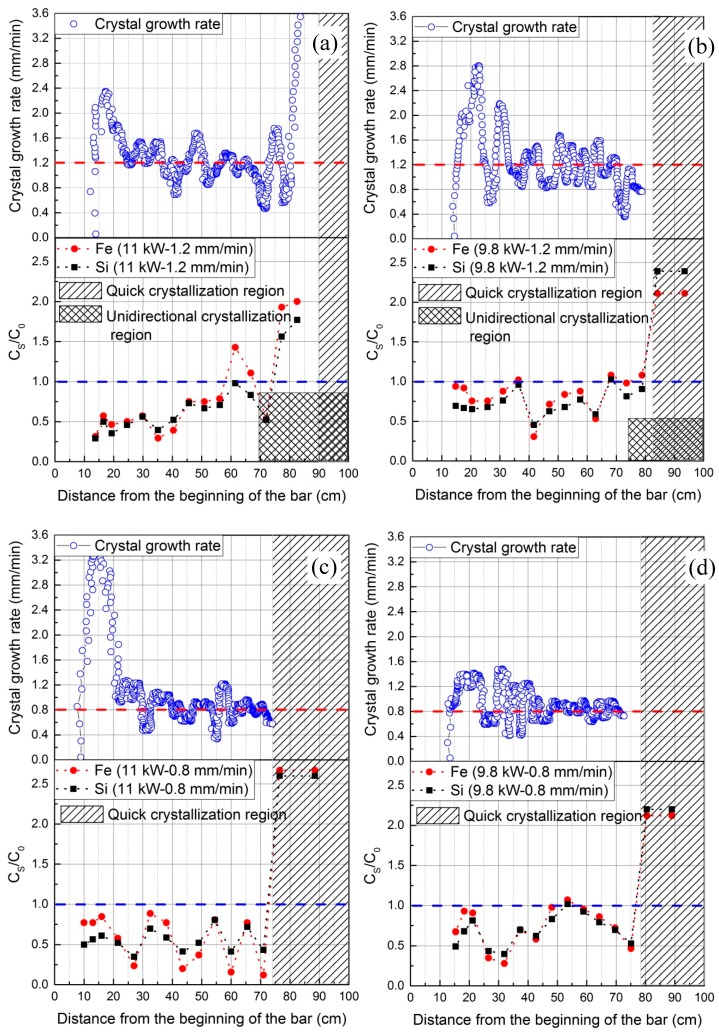
Comparison between the concentration distribution profile of impurities and the crystal growth rate for different experiments: (**a**) 11 kW-1.2 mm/min; (**b**) 9.8 kW-1.2 mm/min; (**c**) 11 kW-0.8 mm/min; (**d**) 9.8 kW-0.8 mm/min.

As mentioned in Section 3.1, the crystal growth rate at every position of freezing interface can be attained by analyzing the data from the IR camera. These results, together with the corresponding impurity distribution profiles, are shown in Figure 11. The crystal growth rate fluctuated constantly around the heater movement velocity (red dashed lines). The fluctuation range was especially large at the beginning of crystallization (around the first 10 cm). This was supposedly because of the thermal loss in this region due to the “cold neighborhood” at the beginning of the bar and shortly before the heating starting area (see Figure 1). This probably led to a fast solidification or a very high crystal growth rate in this area while departing the heating coil. In contrast, Figure 11c shows the lowest fluctuation scope in the region after 10 cm, which indicates that higher power and lower heater movement velocity could result in more stable crystal growth rate.

These phenomena will surely have an influence on refining efficiencies. For all experiments, the high crystal growth rates at the beginning were related to higher impurity concentrations, i.e., lower refining efficiency. Almost each of the peaks or valleys in the crystal growth rate curves corresponded to a drastic increase or decrease in the concentration distribution curves. Surprisingly, a valley takes place at the region of around 60 cm in all cases; the concentration of solids should have continued increasing due to the high accumulation of impurities in the molten zone. This can be explained by the low crystal growth rate that existed in that region. Taking Figure 11a into consideration, when the freezing interface arrived at 50 cm, the crystal growth rate started to decrease. This was probably due to the fact that the heating dissipation was decreasing in that area as the melting interface reached the end of the crucible. However, the mass of graphite involved in the induction was kept the same, meaning the same power input to the molten zone was to be expected. Hence, a heat balance could have taken place at the freezing interface, resulting in its slow movement velocity. In contrast, when the freezing interface passed 57 cm, the melting interface had already arrived at the end of the crucible. Therefore, an increase of crystal growth rate happened, which was possibly due to the sudden disruption of the heat balance. However, the crystal growth rate soon decreased again, which was caused by the participation of the edge mass of graphite in the crucible in the electromagnetic induction and providing additional power. Furthermore, the overall graphite involved in induction sharply decreased, resulting in a drastic increase of crystal growth rate.

In addition, as stated in Section 3.2, a greater zone length can dilute the impurity concentration more extensively, resulting in lower C_L_ and hence lower C_S_ values. However, a clear correlation between impurity concentration profiles and zone length curves cannot be established when observing Figure 9 and Figure 11. This could be due to the offset of the influence of crystal growth rate on refining efficiency. Nevertheless, Figure 11 generally shows that the higher power and lower heater movement velocity (Figure 11c) results in higher refining efficiency, while lower power and lower heater movement velocity (Figure 11b) results in the opposite. This is the joint result of the zone length (affected by power and movement velocity; see in Section 3.2) and the crystal growth rate.

## 4. Conclusions and Outlook

An IR camera has been applied for the first time in zone refining of 2N8-pure aluminum with dopants Fe and Si in order to characterize and interpret the zone refining process. The relationships of the three characteristic positions of freezing/melting interfaces and the heater as well as zone length and crystal growth rate have been derived live through online thermographic analysis based on the temperature information near the molten zone. The influences of heating power and heater movement velocity on zone length variation and crystal growth rate, with respect to only one zone pass, could be derived. Furthermore, interpretations of impurity concentration distribution profiles have been conducted in correlation with the crystal growth rate. The main conclusions drawn are the following:

1. The application of an IR camera is a good way to control and characterize the zone refining process, which can derive the current position of the molten zone, the size of zone length and the actual crystal growth rate online.
2. Zone length varies with the tendency to firstly decrease and then increase for all power and heater movement velocity combinations (see Figure 9). The percentage difference of zone length between maximum and minimum can be depressed by increasing the power. Lowering the heater movement velocity delays the freezing interface position where the zone length becomes minimal.
3. Effective refinement (50% to 75% Fe/Si-reduction) of aluminum happens for all experiments with only one zone pass, but higher power and lower velocity result in the highest refining efficiency. However, the impurity concentration distribution profile after zone refining fluctuates along the bar in each case.
4. Crystal growth rate is not equal to the heater movement velocity and instead, fluctuates around the heater movement velocity. The fluctuations of the impurity concentration profile after refinement mainly results from the variation of crystal growth rate.

The large and unstable variation of zone length and crystal growth rate reveals the challenge to improve the refining efficiency during zone refining of good thermal conductors such as aluminum, especially when using inductive heaters. The realization of an online connection between the infrared camera and the control system of the equipment, which allows for automatization to adjust the power as well as the movement velocity in order to always keep an instant zone length, is strongly recommended for its huge potential to significantly improve refining efficiency.

## Figures and Tables

**Table 1:** Experimental parameters.

Power	Heater Movement Velocity	
11 kW	1.2 mm/min	0.8 mm/min
9.8 kW	1.2 mm/min	0.8 mm/min

**Table 2:** Comparison of characteristic values of zone length variation by changing process parameters.

Characteristic Values	11 kW-1.2 mm/min	9.8 kW-1.2 mm/min	11 kW-0.8 mm/min	9.8 kW-0.8 mm/min
Peak of zone length at the beginning (L_p1_)	28 cm	22 cm	32 cm	28 cm
Peak of zone length at the end (L_p2_)	32 cm	28 cm	26 cm	23 cm
Minimum zone length (L_min_)	24 cm	18 cm	22 cm	19 cm
1-(L_min_/L_p1_)	14.3%	18.2%	31.2%	32.1%
1-(L_min_/L_p2_)	25.0%	35.7%	15.4%	17.4%
Position of freezing interface for L_min_	24 cm	21 cm	49 cm	50 cm
